# Hypophosphatemia in Chronic Obstructive Pulmonary Disease Patients Requiring Mechanical Ventilation and Its Impact on Weaning in an Intensive Care Unit of a Tertiary Care Hospital in Eastern India

**DOI:** 10.7759/cureus.60619

**Published:** 2024-05-19

**Authors:** Amrita R Kundu, Asif Ahmed, Anu Prasad

**Affiliations:** 1 Critical Care Medicine, Tata Main Hospital, Jamshedpur, IND

**Keywords:** respiratory failure, hypophosphatemia, copd, mechanical ventilation, weaning

## Abstract

Background

Hypophosphatemia, defined as a serum phosphate level less than 2.5 mg/dL, is a frequent finding in patients with chronic obstructive pulmonary disease (COPD) and has been speculated to negatively affect weaning outcomes. This study aimed to determine the incidence of hypophosphatemia in COPD patients requiring mechanical ventilation and evaluate the predictive role of hypophosphatemia as an indicator of successful weaning from mechanical ventilation in such patients admitted to the intensive care unit (ICU) in a tertiary care hospital in eastern India.

Methodology

This prospective observational study included 60 adult patients aged 18 to 75 years with acute exacerbations of COPD on mechanical ventilation in the ICU who were planned to undergo a weaning trial. Serum phosphate levels were assessed at the time of admission and before each weaning attempt. Weaning outcomes at each attempt, length of ventilator and ICU stay, and mortality were recorded. Data collection was initiated after approval of the Institutional Ethics Committee. Receiver operating curve (ROC) analysis was done to identify the cut-off value of serum phosphate which predicted successful weaning.

Results

Of 60 participants, hypophosphatemia on admission was present in 15 (25%) patients. Despite the correction, 13 (21.7%) patients had hypophosphatemia before the first weaning attempt. Only 22 patients out of 60 were successfully weaned off from mechanical ventilation in the first trial, accounting for a success rate of 36.7%, of whom 20 were normophosphatemic (90.9%). In the second and third weaning trials, hypophosphatemia was significantly associated with weaning failure. Overall differences in mean serum phosphate levels among those who failed to wean in each weaning trial and the successful attempt were statistically significant (p < 0.001). On ROC analysis of serum phosphate level before the first weaning trial, a cut-off value of ≥3.0 mg/dL was identified to have 86.4% sensitivity, 55.3% specificity, 52.8% positive predictive value, 87.5% negative predictive value, and 66.7% diagnostic accuracy in predicting weaning success. Five patients died, accounting for a mortality rate of 8.3%. Lower mean serum phosphate levels before the first weaning trial, higher mean age, and longer ventilator and ICU days were significantly associated with mortality among our study participants (p < 0.05).

Conclusions

Our findings suggest that maintaining normal serum phosphate levels is critical to successfully weaning off patients with COPD from ventilator support.

## Introduction

Mechanical ventilation is a crucial supportive therapy for maintaining oxygenation in critically ill patients with acute respiratory failure secondary to exacerbation of chronic obstructive pulmonary disease (AECOPD) [1,2]. However, prolonged mechanical ventilation is not usually recommended because it is linked to serious, sometimes fatal, consequences, such as ventilator-associated pneumonia, collapsed lung, and even death [1,3]. Therefore, once the underlying cause of acute respiratory failure has been addressed, weaning off from the ventilator should be attempted. Even so, literary evidence indicates that it is challenging to wean 20% to 30% of patients [4]. Moreover, weaning takes up 40% of a patient’s mechanical ventilation time on average which rises to 59% in chronic obstructive pulmonary disease (COPD) patients [5]. Among the factors that lead to weaning failure, phosphate imbalances, typically hypophosphatemia, have been hypothesized as a potential determinant in the weaning of patients with acute respiratory failure, such as patients with COPD [6].

Sustaining a normal level of serum phosphorus is essential for all cellular functions, including those involved in the physiological processes related to muscular contractility in the body [7,8]. Notwithstanding the paucity of research in this domain [9], it is reasonable to assume that low serum phosphorus levels may be the cause of muscular weakness that exacerbates COPD [10]. Furthermore, a low blood phosphorus level has been reported to exacerbate the severity of COPD, probably through its effect on respiratory muscles, thereby warranting prolonged ventilator support [11,12].

Although there is the availability of literature on the association of hypophosphatemia with critical illness, mainly sepsis, there is limited evidence on its role in patients admitted to the intensive care unit (ICU) with acute exacerbations of COPD, both in terms of weaning outcomes as well as ICU outcomes. Thus, the present study aimed to determine the incidence of hypophosphatemia in COPD patients requiring mechanical ventilation and evaluate whether hypophosphatemia could serve as a predictor of success-to-wean from mechanical ventilation in such patients admitted to the ICU in a tertiary care hospital in eastern India.

This paper was previously presented as a poster at the CRITICARE 2024 Conference held in Kolkata on March 01, 2024.

## Materials and methods

Study design and participants

This prospective observational study was conducted in the Department of Critical Care Medicine, Tata Main Hospital, Jamshedpur. The study included adult patients aged 18 to 75 years with AECOPD on mechanical ventilation in the critical care unit of the study hospital, who met the criteria for weaning, which included patients who were able to breathe spontaneously, had an adequate cough reflex, had respiratory rate <35/minute, did not require vasopressors for blood pressure maintenance, had peak end-expiratory pressure <5 cmH_2_O, had oxygen saturation ≥90% with FiO_2_ <40%, and had rapid shallow breathing index <105. Patient surrogates not providing informed consent; those discharged against medical advice; those suffering from chronic kidney disease; those with a known history of liver disease, heart failure, left ventricular dysfunction with ejection fraction <40%, right ventricular dysfunction, COVID-19 infection, and diabetes mellitus on insulin therapy; and pregnant patients were excluded.

Sample size calculations were based on the standard formula: n = $n = z_{(1-\alpha /2)}^{^{2}}p(1-p)/d^{2}$.

With reference to a study by Mohamed et al. [12], reporting the incidence of hypophosphatemia in patients with acute exacerbations of COPD admitted in the ICU was 64%, and considering a 95% confidence limit with 20% relative error, the sample was approximately 54. Allowing for a 10% dropout rate, the final sample size was calculated to be 60.

Study procedure

Data collection was initiated after obtaining approval from the Institutional Ethics Committee (approval number: TMH/AC/IEC/JUN/064/2021, dated June 23, 2021). Upon admission to the ICU, all patients underwent a severity assessment using the Sequential Organ Failure Assessment (SOFA) scoring system. Baseline characteristics and all vital parameters of the participants with comorbidities, if any, were noted. Patients who had been intubated in the emergency department were continued on mechanical ventilation till the time of the weaning trial approached. Patients who were initially on non-invasive ventilation were intubated and put on mechanical ventilation when they showed an inability to protect the airway (altered sensorium), manage tracheal secretions, worsening of dyspnea, and developed signs of hemodynamic instability despite receiving intravenous fluids and vasoactive agents or developed respiratory muscle fatigue. All patients underwent routine laboratory tests on ICU admission. Baseline serum phosphorus levels were estimated at admission to calculate the incidence of hypophosphatemia (<2.5 mg/dL) and corrected if found deficient. All patients were treated following standard protocols and guidelines. If required, vasoactive agents were used to stabilize the vitals of the patients. Patients who met the weaning criteria were considered eligible for a spontaneous breathing trial (SBT).

The patients lying in the semi-recumbent position, with the head of the bed elevated at an angle between 30° and 45°, were put on pressure support (PS) mode of mechanical ventilation with minimal PS (PS of 9 cmH_2_O, positive end-expiratory pressure of 5 cmH_2_O) and minimal FiO_2_ (<35%). Respiratory rate and tidal volume were noted and minute ventilation and rapid shallow breathing index (RSBI) were calculated after three minutes of settling time from these parameters. The decision to continue SBT was determined by the tolerance of the patient to SBT. “The decision to extubate the patients of both groups was taken based on following common criteria after 60 min SBT: good tolerance to SBT with respiratory rate (RR) <30 cycles/minute, hemodynamic stability (heart rate and blood pressure variability ≤20% of baseline), oxygen saturation ≥90%, and absence of increased work of breathing, conscious level (Glasgow Coma Scale, GCS ≥10), adequate cough reflex and, scanty tracheobronchial secretions” [9]. Those patients who failed SBT were continued on controlled mechanical ventilation and a reattempt at weaning was taken after stabilization, with an interval of at least 48 hours between consecutive weaning attempts. After a third failed attempt, tracheostomy was planned for patients with total ventilator days of at least 14 days, in anticipation of difficult weaning. Morbidity was estimated based on ventilator days and length of hospital stay.

On the morning of the weaning trial, blood samples were sent for assessment of serum phosphate. Based on a predefined weaning protocol, weaning success and failure were evaluated. Irrespective of the weaning outcome, electrolyte imbalances (including hypophosphatemia) were corrected accordingly. Intravenous potassium phosphate (available as 45 mM per 15 mL solution) diluted in sterile water for injection and made to a solution of 45 mL (1 mL = 1 mM) was administered via a syringe pump at 9 mL/hour for five hours under constant monitoring for signs of hyperkalemia. All details were recorded in a predesigned, pretested study proforma.

Data analysis

All data were tabulated using Microsoft Excel 2016 (Microsoft Corp., Redmond, WA, USA) and statistical analysis was done using SPSS version 16.0 (SPSS Inc., Chicago, IL, USA). All continuous variables were compared using the independent t-test (for parametric data) or the Mann-Whitney U test (for non-parametric data). The chi-square test (and Fisher’s exact test, as applicable) was used to check the association between hypophosphatemia and weaning outcome. The receiver operating curve (ROC) was used to identify the cut-off value of serum phosphate for predicting successful weaning. A p-value <0.05 was considered statistically significant.

## Results

**Figure 1:**
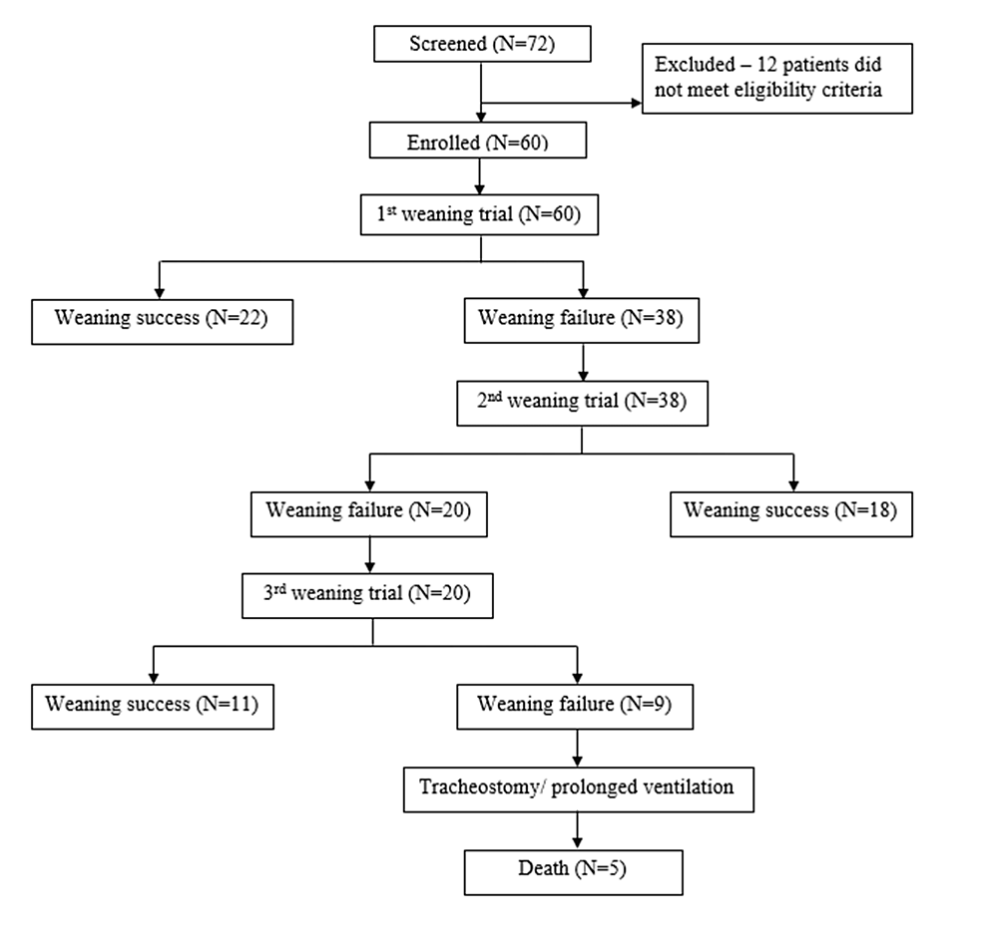
Flowchart depicting the conduct and outcomes of the study.

A schematic representation of the enrolment of study participants, the conduct of the study, and the outcomes of each weaning trial attempt is shown in Figure 1.

Among the 60 patients of AECOPD admitted to the ICU who participated in the study, the mean age was 62.7 (±6.1) years. An almost equal proportion of participants were aged between 55 and 60 years (n = 15, 25%), 60 and 65 years (n = 15, 25%), and 65 and 70 years (n = 16, 26.7%). The minimum age was 49 years, and the maximum age was 75 years. The male-to-female ratio was 1.4:1. Overall, 51 (85%) patients had one or more comorbidities. Moreover, hypophosphatemia on admission was present in 15 (25%) patients (Table 1).

**Table 1:** Baseline characteristics of study participants.

Characteristics	Frequency (%)
Age	
<55 years	5 (8.3)
55–60 years	15 (25.0)
60–65 years	15 (25.0)
65–70 years	16 (26.7)
≥70 years	9 (15.0)
Gender	
Male	35 (58.3)
Female	25 (41.7)
Comorbidity	
Hypertension	36 (60.0)
Diabetes	34 (56.7)
Hypothyroidism	4 (6.7)
No comorbidity	9 (15.0)
Serum phosphate level	
Hypophosphatemia (<2.5 mg/dL)	15 (25.0)
Normophosphatemia (≥2.5 mg/dL)	45 (75.0)

**Figure 2:**
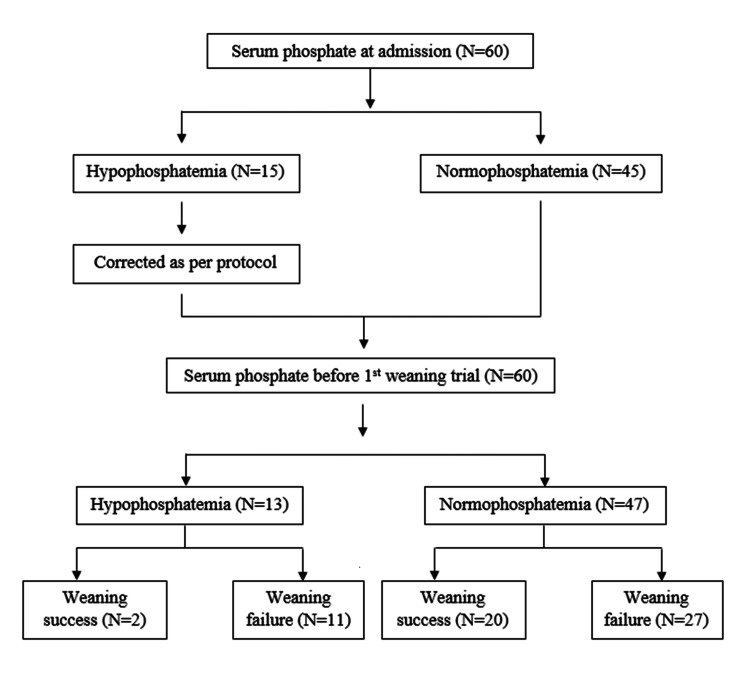
Flowchart showing serum phosphate levels at the baseline and before the first weaning trial with outcomes.

Measurement of serum phosphate levels on the morning of the first weaning trial showed that despite correcting identified hypophosphatemia as per the ICU protocol, 13 (21.7%) patients had hypophosphatemia before the first weaning attempt (Figure 2).

Only 22 out of 60 patients were successfully weaned off from mechanical ventilation in the first trial, accounting for a success rate of 36.7%, of whom 20 were normophosphatemic (90.9%). However, there was no significant association between serum phosphate status and success-to-wean in the first weaning trial (p = 0.072) (Table 2).

**Table 2:** Comparison of serum phosphate levels from mechanical ventilation in AECOPD patients during the weaning trials between weaning outcome groups. Values are presented as n (%) or mean (±SD). ^a^: Seven patients had hypophosphatemia at baseline and six patients developed hypophosphatemia before the first weaning trial. ^b^: Three patients had hypophosphatemia before the first weaning trial and eight patients developed hypophosphatemia before the second weaning trial. ^c^: Four patients had hypophosphatemia before the second weaning trial and five patients developed hypophosphatemia before the third weaning trial. AECOPD = acute exacerbations of chronic obstructive pulmonary disease

Serum phosphorus level	Total	Mean (±SD) phosphate (mg/dL)	Success-to-wean	Failure-to-wean	ꭓ^2^ value	P-value
First weaning trial (N = 60)						
Hypophosphatemia^a^	13 (21.7)	1.96 (±0.48)	2 (9.1)	11 (28.9)	3.237	0.072
Normophosphatemia	47 (78.3)	3.63 (±0.68)	20 (90.9)	27 (71.1)		
Total	60		22	38		
Second weaning trial (N = 38)						
Hypophosphatemia^b^	11 (28.9)	2.04 (±0.17)	1 (5.6)	10 (50.0)	5.185	0.002
Normophosphatemia	27 (71.1)	2.92 (±0.39)	17 (94.4)	10 (50.0)		
Total	38		18	20		
Third weaning trial (N = 20)						
Hypophosphatemia^c^	9 (45.0)	2.43 (±0.74)	0	9 (100.0)	20.000	<0.001
Normophosphatemia	11 (55.0)	2.36 (±0.58)	11 (100.0)	0		
Total	20		11	9		

The patients in the hypophosphatemia group and normophosphatemia groups (as per the first weaning trial) were comparable in terms of baseline demographic characteristics, blood parameters, vital parameters, including GCS and SOFA score at admission, and respiratory parameters (mean respiratory rate, minute ventilation, and RSBI) (p > 0.05). Only, the mean tidal volume was significantly higher in the normophosphatemia group (339.4 ± 54.4 mL) when compared to the hypophosphatemia group (300.9 ± 73.8 mL) (p = 0.04).

The patients with weaning failure in the first trial (n = 38) were subjected to a second weaning trial once they met the predefined weaning criteria. A total of 18 patients were successfully weaned off the ventilator (47.4%). Besides, 11 out of 38 patients (28.9%) were found to have hypophosphatemia before the second weaning attempt, of whom 90.1% had weaning failure; while 10 out of 27 patients with normophosphatemia (37%) had weaning failure. This association was statistically significant (p = 0.002) (Table 2).

The 20 patients in whom the second weaning trial failed, underwent a third weaning trial, where 11 patients had weaning success (55%), and all of these patients were found to have normal phosphate levels. Concomitantly, nine patients faced weaning failure, all of whom were noted to have hypophosphatemia (Table 2).

**Figure 3:**
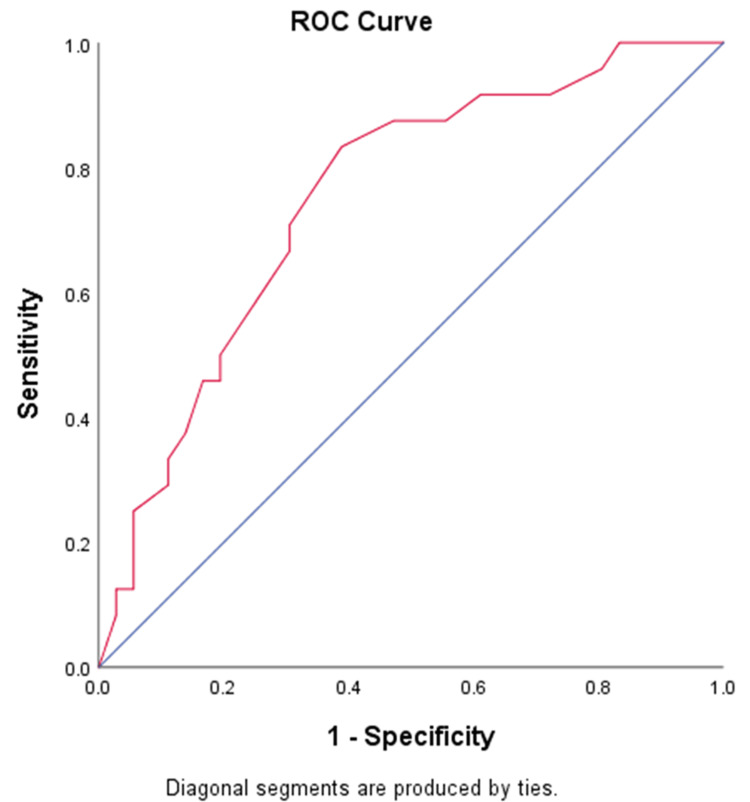
ROC curve for serum phosphate level before the first weaning trial predicting weaning success. ROC = receiver operating curve

ROC analysis of the serum phosphate level before the first weaning trial predicting weaning success had an area under the curve of 0.751, with a cut-off value of ≥3.0 mg/dL identified to have the best sensitivity and specificity in predicting weaning success (Figure 3).

The serum phosphate cutoff value of ≥3.0 mg/dL yielded 86.4% sensitivity, 55.3% specificity, 52.8% positive predictive value, 87.5% negative predictive value, and 66.7% diagnostic accuracy in predicting favorable weaning outcomes.

**Figure 4:**
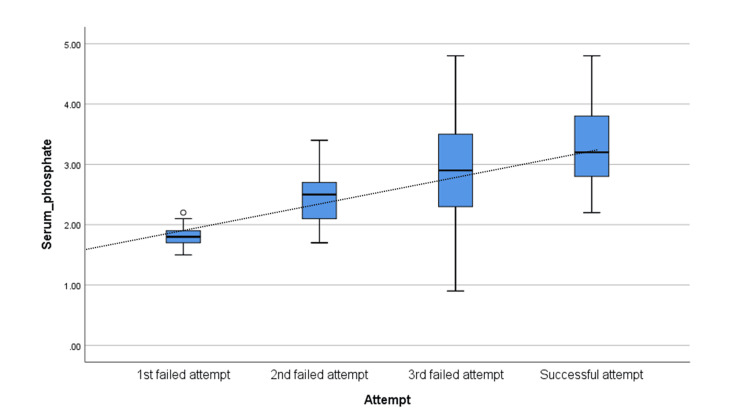
Box plots showing the mean serum phosphate levels according to failed and successful weaning.

Of 60 study participants, at the end of the three weaning trials, 51 patients had weaning success. Overall differences in mean serum phosphate levels among those who had failure-to-wean in each weaning trial and the successful attempt were statistically significant (p < 0.001). The increasing trend, indicated by the dotted trend line, was also significant (p < 0.001) (Figure 4).

Five patients died (due to sepsis and multiorgan dysfunction), accounting for a mortality rate of 8.3%. Among those who expired, the mean serum phosphate level before the first weaning trial was 2.3 ± 1.2 mg/dL, which was significantly lower than those who were discharged (p < 0.001). Moreover, the mean age, ventilator days, and ICU days were significantly higher among the expired patients compared to the discharged patients (p < 0.05) (Table 3).

**Table 3:** Comparison of serum phosphate levels before the first weaning trial, ventilator days, and ICU days according to the final outcome among the study participants. Values are presented as mean ± SD. ICU = intensive care unit

Characteristics	Discharged (N = 55)	Expired (N = 5)	t-value	P-value
Age	65.3 ± 3.8	71.2 ± 1.1	2.081	0.042
Serum phosphate before the first weaning trial	3.4 ± 0.9	2.3 ± 1.2	6.587	<0.001
Ventilator days	6.2 ± 2.6	14.4 ± 3.5	3.143	0.021
ICU days	10.8 ± 3.7	20.4 ± 5.8	3.665	0.019

## Discussion

An exacerbation of COPD is characterized by a worsening of the ongoing respiratory problems, coughing that produces more mucus, and other symptoms that significantly impair daily functioning [11]. The development of respiratory failure, the presence of other chronic conditions, and the requirement for mechanical ventilation during hospitalization all increase the risk of death during a disease flare-up [13]. According to the American Thoracic Society’s International Consensus, 20% of individuals with chronic obstructive lung disease experienced hypophosphatemia [14]. In this study, the incidence of hypophosphatemia among patients with AECOPD on ICU admission was found to be 25%. Our finding is at par with the 18.7% incidence of hypophosphatemia (measured at baseline) reported by Zhao et al. in their patients with AECOPD undergoing weaning from a ventilator [15]. On the contrary, a study by Wang et al. reported a 53.4% incidence of hypophosphatemia in patients with AECOPD admitted to the ICU [16]. These differences in the reported incidence of hypophosphatemia in other studies may be attributed to the study methodology, as the study by Wang et al. was retrospective and included a larger number of patients.

Phosphate levels in ICU patients may fluctuate more quickly than in the non-ICU population, with possibly more dire implications. According to Alsumrain et al., hypophosphatemia affected 21.2% of ICU patients [7]. Brunelli and Goldfarb reported that 28-33% of patients admitted to the ICU were found to have hypophosphatemia [17]. According to Doig et al., hypophosphatemia was observed in 37% of the 2,915 patients evaluated throughout their ICU stay; of these, 50% of instances occurred within the first eight days of ICU admission [18]. In our study, patients with hypophosphatemia at baseline were corrected accordingly. However, serum phosphates measured before the first weaning trial indicated that 21.7% had hypophosphatemia, with a mean serum phosphate of 1.96 ± 0.48 mg/dL; while the value was 3.63 ± 0.68 mg/dL among those with normophosphatemia. As AECOPD patients on mechanical ventilation consistently tend to have low serum phosphorus, it is important to periodically monitor these patients and rectify phosphorus deficiency in accordance with test findings [11].

Our study could not find any significant difference between patients with hypophosphatemia and normophosphatemia before the first weaning trial in terms of age and gender (p > 0.05), which is consistent with that reported by Farah et al. [11].

Weaning should not start until the underlying pathology that requires mechanical breathing has been addressed [7]. In our study, the proportion of weaning failure in the first trial was 84.6% (11 out of 13) among those with hypophosphatemia, while the figure was 57.4% (27 out of 47) among those with normophosphatemia. However, the difference was not significant in the first weaning attempt. On stratified analysis, it was noted that among the patients with normophosphatemia, the proportion of comorbidities was significantly higher among those who failed to wean out of the ventilator compared to those with hypophosphatemia. This could be the reason for a higher percentage of weaning failure rates despite being normophosphatemic.

For subsequent weaning trials, hypophosphatemia was found to be significantly associated with weaning failure. A study by Zhao et al. also reported consistent findings, where the percentage of weaning failure among patients with AECOPD was significantly higher in the hypophosphatemic versus the normophosphatemic groups (p < 0.05) [15]. Although conducted among general ICU patients, Brunelli and Goldfarb demonstrated that adequate serum phosphate level is associated with successful weaning from the ventilator [17].

In this study, the data were analyzed using an ROC curve to determine the optimum cut-off for serum phosphate as a predictor of weaning success. The area under the curve of 0.751 suggests that the association of phosphorus levels with weaning success was strong with a statistical significance (p < 0.001). The cut-off determined from the ROC curve was 3.0 mg/dL, which yielded the best combination of sensitivity, specificity, and diagnostic accuracy in predicting weaning success. Alsumrain et al. reported a cut-off value of 1.05 mmol/L, which is equivalent to 3.3 mg/dL, with phosphates above the cut-off value statistically associated with weaning success [7].

Furthermore, 20 patients required three weaning trials, following which only 11 patients could be successfully weaned off, while the remaining nine patients were difficult to wean and were tracheostomized. Following corrections in electrolyte imbalance, we measured serum phosphate levels before each trial to determine if the patients would be successfully weaned off in subsequent approaches toward an apparently suitable phosphorus level. The median serum phosphorus levels were found to rise significantly as the patients moved toward a successful outcome. The median values of serum phosphorus demonstrated a significant increase (p < 0.001), indicating a statistically higher value of mean serum phosphates among those successfully weaned compared to every failed weaning attempt.

Diaphragmatic weakness, most likely due to low serum phosphate levels, is a recognized cause of mechanical ventilation and weaning failure [13,19]. Because diaphragmatic function improves after recovering from hypophosphatemia, it is strongly encouraged to maintain normal phosphorus levels in patients on mechanical ventilation before ventilator withdrawal [8,19]. Additionally, it makes coughing easier and minimizes the accumulation of secretions in the respiratory system, which could otherwise make ventilated patients more susceptible to infections [20]. Along with hypophosphatemia, which is frequently missed and whose correction might shorten the duration of mechanical ventilation, low levels of potassium and magnesium, starvation, and hypoxia of the respiratory muscles can further delay the weaning process [19].

It needs to be emphasized that, in our study, not all patients who had hypophosphatemia before the first weaning trial, after correction were still hypophosphatemic in the second or third trial. With prolonged ICU stay under sustained mechanical ventilation, accentuated by comorbidities and other electrolyte imbalances, along with increased chances of urinary phosphate excretion in response to commonly prescribed medications such as beta-agonists, diuretics, theophylline, and steroids, patients who were normophosphatemic initially became hypophosphatemic eventually, as assessed before the second and third weaning trial. There were very few patients who remained hypophosphatemic despite corrections.

The mortality rate was 8.3% among our study participants, and all deaths were observed among patients who failed to be weaned off the ventilator after three weaning trials. Mortality in our study was similar to 5% in patients with hypophosphatemia with AECOPD in the ICU, as reported by Farah et al. The factors observed to be associated with mortality in COPD were increased age, hypophosphatemia before the first weaning trial, and a higher duration of ventilator and ICU stay [11]. Congruent with this finding, Shor R et al. found mortality to be significantly associated with severe hypophosphatemia [21]. However, contrary to our study, their conclusions were based on a small sample of sepsis patients as study participants [21].

The strength of the present study lies in its prospective design, which allows for sequential testing before every weaning attempt and subsequent correction. However, this study also had some limitations. Owing to the limited sample size, the results obtained cannot be validated outside the present research, making it difficult to arrive at a definitive conclusion. Further large-scale multicentric studies in different parts of the country may be taken up to validate the findings of the present study.

## Conclusions

The present study is by far the only research conducted in an Indian setting to determine the incidence and role of hypophosphatemia in COPD patients as a predictor of success-to-wean from mechanical ventilation. Based on our observations, we conclude that maintaining normal serum phosphate levels is critical to successfully wean off patients with COPD from ventilator support. It is easy, cheap, and instructive to monitor blood phosphorus levels so that the proper course of treatment can be taken into account. It is also imperative to rectify other electrolyte derangements that come along with hypophosphatemia to ensure a better prognosis in patients with COPD.
